# Influence of the Extraction Method on the Quality and Chemical Composition of Walnut (*Juglans regia* L.) Oil

**DOI:** 10.3390/molecules27227681

**Published:** 2022-11-08

**Authors:** Youssef Elouafy, Adil El Yadini, Hamza El Moudden, Hicham Harhar, Mohammed Merae Alshahrani, Ahmed Abdullah Al Awadh, Khang Wen Goh, Long Chiau Ming, Abdelhakim Bouyahya, Mohamed Tabyaoui

**Affiliations:** 1 Laboratory of Materials, Nanotechnology and Environment LMNE, Faculty of Sciences, Mohammed V University in Rabat, Rabat BP 1014, Morocco; 2 Higher School of Technology of El Kelaa Des Sraghna, Cadi Ayyad University, El Kelaa Des Sraghna BP 104, Morocco; 3 Department of Clinical Laboratory Sciences, Faculty of Applied Medical Sciences, Najran University, P.O. Box 1988, Najran 61441, Saudi Arabia; 4 Faculty of Data Science and Information Technology, INTI International University, Nilai 71800, Malaysia; 5 PAP Rashidah Sa’adatul Bolkiah Institute of Health Sciences, Universiti Brunei Darussalam, Gadong BE1410, Brunei; 6 Laboratory of Human Pathologies Biology, Faculty of Sciences, Mohammed V University in Rabat, Rabat BP 1014, Morocco

**Keywords:** walnut oil, oil extraction techniques, fatty acid profile, quality indices, tocopherols, phytosterols, correlation coefficient, principal component analysis (PCA)

## Abstract

The present study investigated and compared the quality and chemical composition of Moroccan walnut (*Juglans regia* L.) oil. This study used three extraction techniques: cold pressing (CP), soxhlet extraction (SE), and ultrasonic extraction (UE). The findings showed that soxhlet extraction gave a significantly higher oil yield compared to the other techniques used in this work (65.10% with *p* < 0.05), while cold pressing and ultrasonic extraction gave similar yields: 54.51% and 56.66%, respectively (*p* > 0.05). Chemical composition analysis was carried out by GC–MS and allowed 11 compounds to be identified, of which the major compound was linoleic acid (C18:2), with a similar percentage (between 57.08% and 57.84%) for the three extractions (*p* > 0.05). Regarding the carotenoid pigment, the extraction technique significantly affected its content (*p* < 0.05) with values between 10.11 mg/kg and 14.83 mg/kg. The chlorophyll pigment presented a similar content in both oils extracted by SE and UE (*p* > 0.05), 0.20 mg/kg and 0.16 mg/kg, respectively, while the lowest content was recorded in the cold-pressed oil with 0.13 mg/kg. Moreover, the analysis of phytosterols in walnut oil revealed significantly different contents (*p* < 0.05) for the three extraction techniques (between 1168.55 mg/kg and 1306.03 mg/kg). In addition, the analyses of tocopherol composition revealed that γ-tocopherol represented the main tocopherol isomer in all studied oils and the CP technique provided the highest content of total tocopherol with 857.65 mg/kg, followed by SE and UE with contents of 454.97 mg/kg and 146.31 mg/kg, respectively, which were significantly different (*p* < 0.05). This study presents essential information for producers of nutritional oils and, in particular, walnut oil; this information helps to select the appropriate method to produce walnut oil with the targeted quality properties and chemical compositions for the desired purpose. It also helps to form a scientific basis for further research on this plant in order to provide a vision for the possibility of exploiting these oils in the pharmaceutical, cosmetic, and food fields.

## 1. Introduction

Currently, aromatic and medicinal plants have a considerable asset [1] due to the continuous discovery of useful applications of their essential and vegetable oils in the field of health along with their employment in other fields of economic interest [2]. Due to their many uses, they are in ever-increasing demand on world markets. At the national level, Morocco has a variety of aromatic and medicinal plants that can be applied in different fields (pharmacy, perfumery, cosmetics, and food processing) for their therapeutic and fragrant properties [3], and one of the most popular and cultivated plants in Morocco is the walnut (*Juglans regia* L.) plant.

The walnut oils studied in this work are those from the seeds of Azilal province, which are labeled as a geographical indication under the name of «AZILAL WALNUT» [4], and according to the Moroccan Official Bulletin N°6336 offered by the same reference, «AZILAL WALNUT» has a high quality in terms of the physicochemical characteristic of 100 g seeds, with a content of proteins (N × 6.25) ≥ 12 g, lipids ≥ 66 g, carbohydrates ≥ 15 g, and Vitamin B1 (0.15 to 0.18 g) and Vitamin E ≥ 2.5 mg [5], which is what makes it a solid source of nutritional compounds. They are also regarded as oil crops based on their high oil content, which holds various bioactive and health-promoting components, such as tocopherols, phytosterols, chlorophylls, and carotenoids [6], including minor components. Furthermore, walnut oil is rich in polyunsaturated fatty acids (PUFAs), especially linolenic acid, which plays an influential role in coronary heart disease prevention, as well as hypertension and blood lipid regulation [7,8,9]. 

Upon recent reports, walnuts appear to be gaining considerable attention for their beneficial health properties [10], including antidepressant, anti-inflammatory, as well as antidiabetic activities [11], primarily due to the availability of bioactive compounds such as phytosterols and plant polyphenols [12,13,14,15,16]. In addition, consuming walnuts has been associated with many health benefits, as they are an excellent source of omega-3 fatty acids, with a percentage up to 10% [17], vitamin E [18,19,20], as well as other antioxidants that are involved in cardiovascular health and reducing cancer cell growth [21]. 

There are many different factors involved in determining the quality of vegetable oils, from the time the seeds are harvested to the time the oils are consumed, and one of the most important parameters that directly affect the quality of the oil is the extraction method. A reliable oil extraction method is one that can safeguard the quality and preserve more nutrient components in the oil. Many types of extraction techniques exist, including conventional solvent and mechanical extraction [22,23], as well as nonconventional techniques such as supercritical fluid extraction, ultrasonic extraction, microwave extraction, and enzyme-assisted extraction, which have been optimized and applied to improve the oil extraction rates in a shorter time and with minimal deterioration of the oil quality [24,25,26,27].

In the current study, we tried to provide a comparative study between three different extraction techniques: the first one is extraction by the cold press, which is an environment-friendly method that requires less energy [28]; the second method is extraction using the soxhlet system, which is the classical method for solid–liquid extraction; the last extraction method used in this study is the ultrasonic-assisted solvent, and this method has allowed us to obtain high-quality edible oils, with a higher yield and a reduced processing time [29].

The aim of this study was to evaluate the effect of the extraction method (cold press, soxhlet extractor, and ultrasonic extractor), of walnut seeds oil (*Juglans regia* L.), on the physicochemical properties (acid value, free fatty acid value, peroxide value, iodine value, saponification value, K_232_, and K_270_), the content of chlorophyll, carotenoid, sterols, and tocopherols, as well as the fatty acid profile of walnut seed oil.

## 2. Materials and Methods

### 2.1. Plant Materials

Harvesting of walnut (*Juglans regia* L.) seeds was carried out in September 2020, in the regions of Beni Mellal-Khenifra, Morocco, exactly from Agnsou N’Ouargue (31°27′29.5″ N 6°52′58.1″ W). After the harvest of the fruits, they were naturally dried, peeled from the husks, and then shelled manually to obtain the kernel only.

### 2.2. Oil Extraction

***Soxhlet Extractor (SE).*** The soxhlet extraction was carried out from fifty grams of powdered seeds samples for 8 h using n-hexane at 80 °C, with a liquid-to-solid ratio of 5:1, followed by removal of solvent at 50 °C under reduced pressure in a rotary evaporator (Heidolph Hei-VAP Precision motor, Schwabach, Germany).

***Cold Press (CP).*** The cold pressing of the walnut seeds was carried out from fifty grams of crushed walnut seeds using Komet DD 85 G presses (IBG Monforts Oekotec GmbH, Mönchengladbach, Germany) at a temperature of 80 °C, which is the temperature that refers to a heating element that comes into contact with the extraction tube to apply heat to the sample to facilitate the extraction procedure.

***Ultrasonic Extractor (UE).*** The ultrasound extraction was performed from fifty grams of powdered walnut seeds using the ultrasonic processor UP100H (100 W, 30 kHz) from (Hielscher Ultrasound Technology, Teltow, Germany). However, the solvent used in this experiment was n-hexane at 45 °C, with a liquid-to-solid ratio of 5:1, and the duration of the extraction did not exceed 30 min. After the leaching was completed, the mixture (walnut powdered seeds and *n*-hexane) was filtered, and the solvent was eliminated at 50 °C under reduced pressure using a rotary evaporator (Heidolph Hei-VAP Precision motor, Germany).

The three extractions were performed in triplicate, and all extracted oils were immediately filled into brown bottles and stored at 3–5 °C until analyzed.

### 2.3. Physicochemical Quality Parameters

The acid value (AV) and the free fatty acids value (FFA) were determined according to the ISO 660 norm [30]; the peroxide value was calculated according to the ISO 3960 [31]; the saponification, the iodine, and the specific extinction coefficients values (K_232_ and K_270_) were determined according to the American Oil Chemists’ Society (AOCS) recommended practice, respectively, Cd 3a-94, Cd 1c-85, and Ch 5-91 [32]. The FFA content reflects the amount of free oleic fatty acid; it is expressed in mg KOH/g oil of free oleic acid in relation to the total amount of the oil; the specific extinction coefficients (K_232_ and K_270_) were expressed as the specific extinction of a 1% (*w*/*v*) solution of the oil in cyclohexane in a 1 cm cell way length, utilizing an (LLG-uniSPEC 2) UV spectrometer. Saponification and iodine values were expressed in (mg KOH/g oil) and (mg of I_2_/100g oil), respectively.

### 2.4. Chlorophylls and Carotenoids Content

The determinations of chlorophylls and carotenoids were carried out by the preparation of a 1% solution of walnut seeds oil in cyclohexane followed by measuring the absorbance at 670 nm and 470 nm, respectively [33]. Contents were calculated using the following formulas, Equations (1) and (2).

(1) $$Chlorophyll\;(mg.kg^{-1})=\frac{A_{670}\times 10^{6}}{613\times 100\times d}$$

(2) $$Carotenoid\;(mg.kg^{-1})=\frac{A_{470}\times 10^{6}}{2000\times 100\times d}\;$$

where *A* is the absorbance and *d* is the thickness of the spectrophotometer cell (1 cm). The chlorophyll and carotenoid content were expressed in ppm, (mg predominantly pheophytin a by kilogram of oil) and (mg Lutein by kilogram of oil), respectively.

### 2.5. Fatty Acids’ Composition

Saturated (SFAs), monounsaturated (MUFAs), and polyunsaturated fatty acids (PUFAs) were determined following the recommended practices required by the American Oil Chemists’ Society (AOCS), Ce 1i-07 [32]. The fatty acids (FAs) underwent a transesterification reaction with methanol to obtain the fatty acid methyl esters (FAMEs). The latter were analyzed on a gas chromatography with capillary column (Varian CP-3800, Varian Inc. Middelburg, The Netherlands) that has a polar stationary phase and split inlet system. The separation was according to the fatty acids chain length (CL), degree of unsaturation, and position of double bonds (which means the position isomers). C17:0 was used as an internal standard for quantification. A fused silica column was used with the following dimensions (l = 30 m; Ø = 0.32 mm). The carrier gas used was helium and the initial and final column temperatures were 170 °C and 230 °C, respectively, with a rising rate of 3 °C/min. The injected volume was 1 μL and the detection was with a flame-ionization detector (FID) at 230 °C. Results are presented as relative percentages of individual fatty acids within the sample.

### 2.6. Phytosterols Composition

The quantification of sterols composition was carried out according to the AOCS Official Method Ch 6-91 [32], which necessitates the saponification of each oil sample, followed by the extraction of the unsaponifiable fraction, then separating sterols in this fraction by gas chromatography with a capillary column (Varian CP-3800, Varian Inc. Middelburg, The Netherlands). 5α-cholestanol was used as an internal standard for quantification. The column dimensions were (l = 30 m, Ø = 0.32 mm), helium was used as a carrier gas, and the injection volume was 1 μL for each analysis. The results of this method can be used to verify the purity of the fat or to check compliance with a commercial denomination or an advertised composition.

### 2.7. Tocopherols Composition

The amounts of tocopherol were quantified following the ISO 9936 standard method using an HPLC equipped with a fluorometric detector on a silica column (25 cm × 4 mm). An isooctane: isopropanol (99%: 1%) mixture was used as an eluent with a rate of 1.2 mL/min for 20 min. Furthermore, the quantification was carried out with the help of external standard curves of α-, β-, γ-, and δ-tocopherols and a quantitative and qualitative daily reference of tocopherol standards [34].

### 2.8. Statistical Analysis

IBM SPSS Statistics 26 software was used to affect the ANOVA One-Way Tukey HSD test for the verification of the statistical significance at a confidence level of 95.0%, and the results were expressed as means ± standard error of the mean (Mean ± SE). Correlation analysis was carried out by Pearson’s test using XLSTAT 2021, and the data obtained were analyzed using principal component analysis (PCA).

## 3. Results and Discussion

### 3.1. Oil Yields and Physicochemical Quality Parameters

The yield of the oils obtained by each of the extraction methods, SE, CP, and UE, was calculated using Equation (3).

(3) $$Oil\;yeild=\frac{weight\;of\;extracted\;oil\;(g)}{weight\;of\;walnut\;seed\;(g)}\times 100$$

SE was the most suitable technique to improve the oil yield compared to the others, yielding 65.10 ± 2.13%, followed by CP and UE, which gave similar yields of 54.51 ± 2.46% and 56.66 ± 1.97%, respectively (*p* > 0.05). It can be said that the extraction technique had an impact on the oil yield, SE was more efficient than the other investigated methods, and these results are similar to those of other studies, which reported that solvent extraction is more effective for lipid yield from walnut seeds [35,36]. Although hexane extraction is the most efficient in terms of yield, this solvent is highly flammable and can create environmental and health problems [37,38], compared to safer and more environmentally friendly alternatives such as aqueous, enzymatic [39], and supercritical fluid extractions [40], which preserve the quality and are bioactive oil compounds.

In Table 1, we sum up the contents of acid value, free fatty acids, peroxide value, iodine value, saponification value, chlorophyll, carotenoid, K_232_, and K_270_ present in walnut seeds oils, which were extracted using our three-extraction method. As shown, the acid values ranged from 1.52 to 3.90 (mg KOH/g of oil) and, from those results, we calculated the amount of free fatty acids expressed in percentage of oleic acid (C18:1). This parameter can give us an indication of the quality and edibility of the oils [41]. The oil extracted by the CP had the lowest value of FFA% (0.76% of oleic acid) in comparison to the other extraction methods oils (1.07% of oleic acid for UE oil and 1.95% of oleic acid for SE oil), and from this, we can say that the oil produced by cold pressing has the finest quality in terms of acidity, which explains its continued supremacy in popularity among consumers [42]. This does not diminish in any way the value and quality of the other oils, because the three oils did not exceed the maximum limit of 4.0 mg KOH/g of oil according to the Codex Alimentarius Commission [43].

The peroxide value provides a quantitative measurement of the level of primary oxidation products (hydroperoxides) in oils and fats [44]. A peroxide value of 15 (meq O_2_.kg^−1^) is the highest limit of an acceptable oil quality [45]. It clearly appeared that all the three methods provided decent oils in terms of hydroperoxides levels. All values were under the acceptance limits and approximately equal, between 4.14 and 5.12 meq O_2_.kg^−1^, and the highest value came from SE oil. We may attribute this to the high temperature used in this process compared with CP and US in which we did not extract the oils using a raised temperature.

The iodine and saponification values of the oils extracted by the three different methods were almost identical (*p* > 0.05); therefore, it can be said that the extraction method did not influence these parameters. The high iodine values reported in all the oils studied are directly related to the levels of PUFAs [46], as they are more sensitive to oxidative degradation, which leads to producing oils with high iodine values.

The saponification value (SV) is defined as the number of mg of KOH needed to saponify 1 gram of oil [47]. The results found in CP, SE, and UE oils were 189.0, 190.18, and 191.42 mg KOH/g, respectively (*p* > 0.05). These values indicate a higher molecular weight of fatty acid in the triglyceride [48] and it can be one of the biggest reasons why these oils are suitable for inclusion in numerous healthcare products [46].

The specific extinction coefficient at λ = 232 nm (K_232_) determines the peroxides that exist within the hydroperoxide conjugated double bonds, and both CP and UE oils had nearly similar values of K_232_, 1.30 for the CP oil and 1.77 for the UE oil, while the SE oil had the higher value, which was 2.73 (Table 1). The K_232_ and K_270_ values were both higher in the SE oil than they were in the CP and UE oils; the values obtained were 2.49, 0.12, and 0.96, respectively; those values are supported by the results obtained by Grilo et al. in 2021 [44]. It can be said that the SE extraction method provides an oil with a high ability of forming peroxides and degrading them, which can be attributed to the length of the extraction time and/or the temperature used on it.

### 3.2. Chlorophyll and Carotenoid Content

The chlorophyll pigment is mainly used to identify the pro-oxidizing action of the oils, with a content lower than 0.5 mg/kg (Table 1) of our three oils, which varies from 0.13 to 0.20 mg/kg. These low contents are desired to avoid the pro-oxidizing action of the chlorophyll pigments and to ensure a good conservation of the oils [49,50]. It appeared clearly that CP oil had the lowest value compared with the other oils extracted by the other methods implemented in this study with 0.13 ppm, so we can say that the cold press continues to win this comparative bet. Additionally, the carotenoid values found at the oils extracted by CP, SE, and UE were 14.83, 10.11, and 12.46 mg/kg, respectively. Carotenoids are associated with several health benefits, particularly in reducing the potential for chronic diseases such as cardiovascular and eye degenerative diseases [51,52]. It can be seen that the cold pressing technique (CP) offered an oil rich with this pigment, followed by UE and then SE oil coming in last.

### 3.3. Fatty Acid Composition

Table 2 regroups the percentage of fatty acids present in our three oils that were analyzed using capillary gas chromatography and compared to the standards offered by Codex Alimentarius Commission [43].

In this study, eleven fatty acids were recognized, and they consisted of a mixture of polyunsaturated, monounsaturated, and saturated fatty acids with similar percentages. The percentages of PUFAs were as follows: 72.35% for CP, 69.37% for SE, and 71.92% for UE. Based on these results, we can state that the extraction technique did not have a great impact on the PUFA composition. The same trends apply to MUFAs and SFAs, which were also not affected by the extraction techniques.

Regarding saturated fatty acids (SFAs), the results (Table 2) showed that palmitic acid (C16:0) was the most relevant saturated fatty acid, with the highest value of 7.89% which was identical for both oils extracted by the soxhlet and the ultrasonic extractors, and it was 7.76% for the cold-pressed oil. The second relevant SFA was stearic acid (C18:0) with 2.88% in the UE oil and 2.52% in the CP and the SE oils. All other saturated fatty acids were present with similar values in the three different oils with 0.09% for arachidic acid (C20:0), 0.04% for heptadecanoic acid (C17:0), and 0.03% for myristic acid (C14:0). Concerning monounsaturated fatty acids (MUFAs), the results showed that oleic acid (C18:1) was the main monounsaturated fatty acid with a percentage of 16.83% in the cold-pressed oil, 16.80% in the oil extracted by ultrasonic extraction, and 16.38% in the oil extracted by soxhlet extraction. Thereafter, we found palmitoleic acid (C16:1) with a percentage of 0.21% for the UE oil, 0.19% for the SE oil, and 0.18% for the CP oil; finally, gadoleic (C20:1) and heptadecenoic acid (*cis*-10) (C17:1) arrived at the last row with similar percentages in all the three oils, 0.13% and 0.04%, respectively. It can be said that the oleic and palmitoleic acid contents were slightly dependent on the extraction method, while no dependence was detected for the other monounsaturated fatty acids (C20:1 and C17:1). Linoleic acid (C18:2) was the major polyunsaturated fatty acid, and it was highly present in the CP oil with a percentage of 57.84%, followed by the SE oil, with a percentage of 57.58%, and then the UE oil, with a percentage of 57.08%.

The walnut oil fatty acid composition is presented in Table 2 in accordance with the composition reported in previous studies [53,54]. The further investigation conducted by Mariod et al. in 2010 showed that the extraction technique had no impact on the fatty acid composition of *Annona squamosa* and *Catunaregam nilotica* seed oils [55].

It is noted that all the fatty acids (SFAs, MUFAs, and PUFAs) percentages listed in this investigation are in conformity with the standards provided by the Codex Alimentarius Commission [43].

### 3.4. Phytosterol Compositions

The phytosterol compositions of our oils extracted by CP, SE, and UE were analyzed by gas chromatography with a capillary column, which permitted a successful separation for cholesterol, campesterol, stigmasterol, clerosterol, β-sitosterol, Δ^5^-avenasterol, Δ^7^-stigmasterol, and Δ^7^-avenasterol, as shown in Table 3.

The total phytosterol contents of the walnut oils extracted by our three different methods ranged from 1168.55 mg.kg^−1^ for the oil extracted by UE, to 1306.03 mg.kg^−1^ for the cold-pressed oil, while the SE oil reported a content of 1171.29 mg.kg^−1^. Among the phytosterols, β-sitosterol, Δ^5^-avenasterol, and campesterol were in the highest amounts, exceeding 95% in all samples. However, the phytosterol amounts presented in Table 3 are similar to those reported in previous studies [16,56]. β-sitosterol was the dominant phytosterol in all three oils, with a content of (1113.52 mg.kg^−1^) in CP oil, higher than those of the other oils extracted by SE and UE (1005.79 mg.kg^−1^ and 989.18 mg.kg^−1^, respectively). It can be said that the extraction method had an impact on the phytosterol composition, and the cold press extraction gave the richest oil in terms of phytosterols, which can be attributed to the low temperature used in this process [57].

### 3.5. Tocopherol Compositions

The results of α, γ, and δ-tocopherol contents of CP, SE, and UE walnut (*Juglans regia* L.) oils are shown in Table 4.

The table shows that α-tocopherol was not detected in UE walnut oil and that the content of the said isomer varied between 7.40 mg/kg in SE oil and 15.75 mg/kg in CP oil, while the content of γ-tocopherol varied between 128.32 mg/kg in UE oil and 754.83 mg/kg in CP oil, whereas the content of δ-tocopherol varied between 17.99 mg/kg in the oil extracted by UE and 81.06 mg/kg in the cold-pressed oil. The highest content of tocopherol and its isomers in walnut (*Juglans regia* L.) oil was detected in CP oil as γ-tocopherol with an amount of 754.83 mg/kg, while the lowest content was α-tocopherol in SE oil with 7.40 mg/kg, and these results are comparable to those obtained by [58] and [59], which also reported that γ-tocopherol represents the major tocopherol in walnut (*Juglans regia* L.) oil, followed by δ-tocopherol and α-tocopherol, without detection of β-tocopherol or presence in trace amounts.

A further study was conducted by Gharibzahedi in 2013, which also revealed similar results in terms of tocopherol compositions, but within the present paper, higher γ-tocopherol contents were reported in the cold-pressed oil (754.83 mg/kg), compared to (349 mg/kg) in Gharibzahedi’s study [54]. In addition, walnut oil showed a higher total tocopherol content than what was found in *Persea Americana*, *Prunus Amygdalus dulcis*, and *Prunus Amygdalus amarus* oils [45,60].

It is now clear that the extraction technique has a direct impact on the tocopherol content and that the oil obtained by the CP technique represents the highest total tocopherol content with 851.64 mg/kg, followed by the SE and UE oils with 454.97 mg/kg and 146.31 mg/kg, respectively. It is noteworthy that the tocopherols and their isomers have a very important antioxidant power and can, therefore, play an important role in the control or prevention of pre-diabetes, insulin, and vascular lesions [41,61,62].

### 3.6. Principal Component Analysis (PCA)

**Figure 1:**
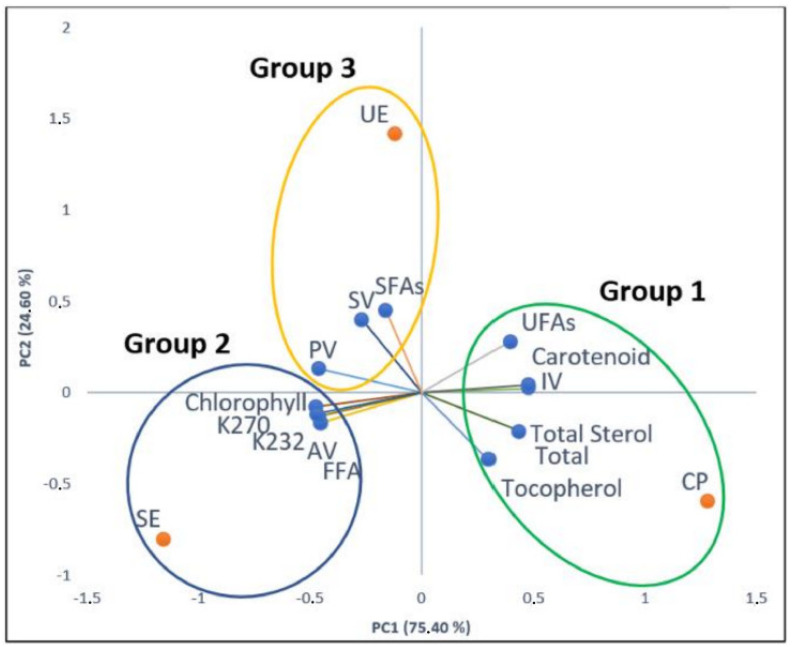
PCA score plot of the first two principal components (PC1 (75.40%); PC2 (24.6%)) of walnut seed oils extracted by different methods.

In our statistical assessment, we used the three replicates (*n* = 3) approach; the three-time repeated analyses were finding the percentages of the saturated and the unsaturated fatty acids (SFAs and UFAs, respectively), the physicochemical properties, the total sterol, as well as the total tocopherol content. The PCA score plot of the first two components of the walnut (*Juglans regia* L.) oils obtained by our three extraction methods is presented in Figure 1. We note that the first two principal components (PC1; PC2) explained 100% of the total variability (Figure 1); PC1 presented 75.40% of the total variance of the data, while PC2 presented 24.60% of the total variance of the data (Table 5).

Table 6 represents Pearson’s correlation matrix coefficient between the physicochemical characteristics and the chemical composition of walnut (*Juglans regia* L.) oils with a significance level of alpha = 0.05. We can clearly see a high correlation between (AV/K_232_), (FFA/K_232_), (IV/carotenoid), as well as (AV/FFA), and this can also be seen in Figure 1. All said compositions and parameters that correlate with each other are located in the same group near each other. In addition to that, Figure 1 gives us a visualization of the entire study by grouping all the analyses carried out in this investigation into three groups, with each group related to a different extraction method. Group 1 related to CP, group 2 related to SE, and group 3 related to UE. On this basis, it can be clearly seen that cold pressing extraction is the most suitable technique to extract phytosterols and tocopherols in higher quantities compared to the other studied techniques, and also preserves the unsaturated fatty acid composition of the walnut oil from degradation by high temperatures.

The results obtained from this study proved that the extraction technique of walnut (*Juglans regia* L.) seeds oil can affect the quality of the extracted oils alongside their fatty acids, phytosterol, and tocopherol percentages.

## 4. Conclusions

The current study provides an evaluation of the effect of extraction method (cold press, soxhlet, and ultrasonic extraction) of Moroccan walnut oil on the fatty acid profile, physicochemical properties, chlorophyll, carotenoid, sterol content, as well as tocopherol compositions of walnut oil. The results showed that *Juglans regia* L. oil is a rich source of unsaturated fatty acids, sterol, and tocopherol (Vitamin E), and that linoleic acid (C18:2) is the main fatty acid and that β-sitosterol is the predominant sterol, while γ-tocopherol is the primary isomer of tocopherol. Ultrasonic extraction may be a time- and solvent-saving technique, but it provides oil with a higher PV content compared to what cold pressing produces. Similarly, the extraction by soxhlet gives the highest yield of oil but also with the highest values of AV and extinction coefficients, in addition to traces of hexane that can be harmful to the health of the consumer. We can conclude that the extraction technique has a direct impact on the content of the composition in phytosterols, as well as tocopherols, and that the cold pressing technique was the most effective method to protect the studied compositions from degradation during the extraction process. Overall, this study presents essential information for producers of nutritional oils and, in particular, walnut *(Juglans regia* L*.)* oil; this information helps to produce a safe walnut oil with high nutritional value using an eco-friendly technique. It also helps to form a scientific basis for further research on this plant in order to provide a vision for the possibility of exploiting these oils in the pharmaceutical, cosmetic, and food fields.

## Figures and Tables

**Table 1:** Physicochemical properties, and chlorophyll and carotenoid content of walnut seed oils extracted by different methods (Mean ± SE).

	CP	SE	UE
**AV (mg KOH/g)**	1.52 ± 0.09 ^a^	3.90 ± 0.02 ^b^	2.13 ± 0.06 ^c^
**FFA (%)**	0.76 ± 0.11 ^a^	1.95 ± 0.02 ^b^	1.07 ± 0.03 ^a^
**PV (meq O_2_/kg)**	4.14 ± 0.09 ^a^	5.12 ± 0.34 ^b^	4.95 ± 0.27 ^b^
**IV (mg I_2_/100 g)**	152.27 ± 0.13 ^a^	151.45 ± 0.17 ^a^	151.83 ± 0.11 ^a^
**SV (mg KOH/1 g)**	189.0 ± 0.12 ^a^	190.18 ± 0.23 ^a^	191.42 ± 0.33 ^a^
**Chlorophyll**	0.13 ± 0.01 ^a^	0.20 ± 0.02 ^b^	0.16 ± 0.01 ^b^
**Carotenoid**	14.83 ± 0.61 ^a^	10.11 ± 0.43 ^b^	12.46 ± 0.56 ^c^
**K_232_**	1.30 ± 0.05 ^a^	2.73 ± 0.07 ^b^	1.77 ± 0.05 ^c^
**K_270_**	0.12 ± 0.02 ^a^	2.49 ± 0.08 ^b^	0.96 ± 0.02 ^c^

Means of three determinations ± Standard Error (Mean ± SE). Values in the same row with different superscript letters are significantly different (*p* < 0.05) according to ANOVA One-Way Tukey HSD test.

**Table 2:** Fatty acid compositions of walnut seed oils extracted by different methods (Mean ± SE).

	CP (%)	SE (%)	UE (%)	Std (%)
**Myristic acid (C14:0)**	0.03 ± 0.01 ^a^	0.03 ± 0.01 ^a^	0.03 ± 0.01 ^a^	ND
**Palmitic acid (C16:0)**	7.76 ± 0.11 ^a^	7.89 ±0.13 ^a^	7.89 ±0.08 ^a^	6.0–8.0
**Palmitoleic acid (C16:1)**	0.18 ±0.09 ^a^	0.19 ±0.03 ^a^	0.21 ±0.07 ^a^	ND–0.4
**Heptadecanoic acid (C17:0)**	0.04 ± 0.01 ^a^	0.04 ± 0.01 ^a^	0.04 ± 0.01 ^a^	ND–0.1
**Heptadecenoic acid (*cis*-10) (C17:1)**	0.02 ± 0.01 ^a^	0.02 ± 0.01 ^a^	0.02 ± 0.01 ^a^	ND–0.1
**Stearic Acid (C18:0)**	2.52 ± 0.06 ^a^	2.52 ± 0.07 ^a^	2.88 ±0.09 ^a^	1.0–3.0
**Oleic Acid (C18:1)**	16.83 ±0.21 ^a^	16.38 ± 0.18 ^a^	16.80 ±0.15 ^a^	14.0–23.0
**Linoleic Acid (C18:2)**	57.84 ±0.18 ^a^	57.58 ±0.10 ^a^	57.08 ± 0.09 ^a^	54.0–65.0
**Linolenic Acid (C18:3)**	15.11 ± 0.10 ^a^	14.51 ±0.12 ^b^	14.84 ±0.07 ^a,b^	9.0–15.4
**Arachidic acid (C20:0)**	0.09 ± 0.04 ^a^	0.09 ±0.07 ^a^	0.09 ± 0.03 ^a^	ND–0.3
**Gadoleic Acid (C20:1)**	0.13 ± 0.02 ^a^	0.13 ± 0.03 ^a^	0.13 ± 0.03 ^a^	ND–0.3
**(SFAs)**	**10.44**	**10.57**	**10.93**	
**(MUFAs)**	**17.16**	**16.72**	**17.61**	
**(PUFAs)**	**72.35**	**69.37**	**71.92**	
**(M/S)**	**1.64**	**1.58**	**1.57**	
**(P/S)**	**6.93**	**6.88**	**6.58**	

(SFAs): Saturated Fatty Acids; (MUFAs) Monounsaturated Fatty Acids; (PUFAs) Polyunsaturated Fatty Acids; (M/S) Monounsaturated fatty acid/Saturate fatty acid ratio; (P/S) Polyunsaturated fatty acid/Saturate fatty acid ratio; ND: Not Detected ≤ 0.05%. Means of three determinations ± Standard Error (Mean ± SE). Values in the same row with different superscript letters are significantly different (*p* < 0.05) according to ANOVA One-Way Tukey’ HSD test.

**Table 3:** Phytosterol Compositions (mg/kg and %) of Oil Extracted from Walnut Samples (Mean ± SE).

	CP		SE		UE	
	mg/kg	%	mg/kg	%	mg/kg	%
**Cholesterol**	5.35 ± 0.17 ^a^	0.41 ± 0.12 ^a^	4.33 ± 0.14 ^b^	0.37 ± 0.10 ^a^	4.44 ± 0.13 ^b^	0.38 ± 0.09 ^a^
**Campesterol**	68.31 ± 0.13 ^a^	5.23 ± 0.09 ^a^	58.10 ± 0.11 ^b^	4.96 ± 0.08 ^a^	58.31 ± 0.10 ^b^	4.99 ± 0.07 ^a^
**Stigmasterol**	3.27 ± 0.06 ^a^	0.25 ± 0.04 ^a^	1.99 ± 0.04 ^b^	0.17 ± 0.03 ^a^	3.86 ± 0.03 ^c^	0.33 ± 0.02 ^a^
**Clerosterol**	8.36 ± 0.14 ^a^	0.64 ± 0.10 ^a^	7.84 ± 0.10 ^a^	0.67 ± 0.07 ^a^	6.78 ± 0.08 ^b^	0.58 ± 0.06 ^a^
**β-Sitosterol**	1113.52 ± 0.17 ^a^	85.26 ± 0.12 ^a^	1005.79 ± 0.16 ^b^	85.87 ± 0.11 ^a^	989.18 ± 0.11 ^c^	84.65 ± 0.08 ^a,b^
**Δ^5^-Avenasterol**	83,72 ± 0.07 ^a^	6.41 ± 0.05 ^a^	74.61 ± 0.08 ^b^	6.37 ± 0.06 ^a^	73.62 ± 0.06 ^c^	6.30 ± 0.04 ^a^
**Δ^7^-Stigmasterol**	0.91 ± 0.04 ^a^	0.07 ± 0.03 ^a^	1.05 ± 0.06 ^a^	0.09 ± 0.04 ^a^	16.83 ± 0.06 ^b^	1.44 ± 0.04 ^b^
**Δ^7^-Avenasterol**	1,57 ±0.07 ^a^	0.12 ± 0.05 ^a^	0.82 ± 0.04 ^b^	0.07 ± 0.03 ^a^	1.64 ± 0.03 ^a^	0.14 ± 0.02 ^a^
**Total (mg/Kg)**	1306.03 ± 0.16 ^a^		1171.29 ± 0.30 ^b^		1168.55 ± 0.20 ^c^	

Values in the same row with different superscript letters are significantly different (*p* < 0.05) according to ANOVA One-Way Tukey HSD test.

**Table 4:** Tocopherols Compound Context (mg/kg and %) of walnut seed oils (Mean ± SE).

	α-Tocopherol		γ-Tocopherol		δ-Tocopherol		Total
	mg/Kg	%	mg/Kg	%	mg/Kg	%	mg/Kg
**CP**	15.75 ± 0.20 ^a^	1.85 ± 0.09 ^a^	754.83 ± 0.14 ^a^	88.63 ± 0.08 ^a^	81.06 ± 0.06 ^a^	9.52 ± 0.02 ^a^	851.64 ± 0.28 ^a^
**SE**	7.40 ± 0.13 ^b^	1.63 ± 0.02 ^a^	408.90 ± 0.11 ^b^	89.87 ± 0.05 ^b^	38.66 ± 0.14 ^b^	8.50 ± 0.06 ^b^	454.96 ± 0.38 ^b^
**UE**	ND	ND	128.32 ± 0.08 ^c^	87.71 ± 0.04 ^c^	17.99 ± 0.20 ^c^	12.30 ± 0.09 ^c^	146.31 ± 0.12 ^c^

Means of three determinations ± Standard Error (Mean ± SE). Values in the same column with different superscript letters are significantly different (*p* < 0.05) according to ANOVA One-Way Tukey HSD test.

**Table 5:** Discrimination table for PCA models constructed with physicochemical properties and the chemical composition of walnut (*Juglans regia* L.) oils.

	F1	F2
**Eigenvalue**	9.802	3.198
**Variability (%)**	75.402	24.598
**% Cumulative**	75.402	100.000

**Table 6:** Pearson’s correlation matrix coefficient between physicochemical characteristics and chemical composition.

Variables	AV	FFA	PV	IV	SV	Chlorophyll	Carotenoid	K_232_	K_270_	T. Sterol	T. Tocopherol	SFA	UFA
**AV**	**1**												
**FFA**	**1.000**	**1**											
**PV**	0.807	0.806	**1**										
**IV**	−0.951	−0.951	−0.950	**1**									
**SV**	0.235	0.234	0.764	−0.524	**1**								
**Chlorophyll**	0.982	0.982	0.904	−0.992	0.414	**1**							
**Carotenoid**	−0.963	−0.962	−0.937	**0.999**	−0.490	−0.996	**1**						
**K_232_**	**0.997**	**0.997**	0.848	−0.971	0.306	0.993	−0.980	**1**					
**K_270_**	0.994	0.994	0.864	−0978	0.336	0.996	−0.986	**1.000**	**1**				
**T. Sterol**	−0.687	−0.686	−0.984	0.878	−0.868	−0.812	0.858	−0.739	−0.760	**1**			
**T. Tocopherol**	−0.318	−0.317	−0.817	0.595	−0.996	−0.491	0.563	−0.387	−0.416	0.907	**1**		
**SFA**	−0.013	−0.014	0.581	−0.297	0.969	0.176	−0.258	0.061	0.092	−0.717	−0.944	**1**	
**UFA**	−0.967	−0.968	−0.630	0.841	0.019	−0.902	0.862	−0.946	−0.935	0.480	0.067	0.266	**1**

(T. Sterol): Total Sterol; (T. Tocopherol): Total Tocopherol. Values in bold are different from 0 at significance level alpha = 0.05.

## Data Availability

All data were included in the manuscript.
